# Biochar and Nitrogen Fertilizer Change the Quality of Waxy and Non-Waxy Broomcorn Millet (*Panicum miliaceum* L.) Starch

**DOI:** 10.3390/foods12163009

**Published:** 2023-08-09

**Authors:** Miaomiao Zhang, Bauyrzhan Mukhamed, Qinghua Yang, Yan Luo, Lixin Tian, Yuhao Yuan, Yani Huang, Baili Feng

**Affiliations:** State Key Laboratory of Crop Stress Biology for Arid Areas, College of Agronomy, Northwest A&F University, Yangling, Xianyang 712100, Chinayuanyuhao@nwafu.edu.cn (Y.Y.);

**Keywords:** broomcorn millet, starch, biochar, nitrogen fertilizer, quality

## Abstract

The overuse of nitrogen fertilizers has led to environmental pollution, which has prompted the widespread adoption of biochar as a soil conditioner in agricultural production. To date, there has been a lack of research on the effects of biochar and its combination with nitrogen fertilizer on the quality of broomcorn millet (*Panicum miliaceum* L.) starch. Thus, this study examined the physicochemical characteristics of starch in two types of broomcorn millet (waxy and non-waxy) under four different conditions, including a control group (N0), nitrogen fertilizer treatment alone (N150), biochar treatment alone (N0+B), and a combination of biochar and nitrogen fertilizer treatments (N150+B). The results showed that, in comparison to the control, all the treatments, particularly N150+B, decreased the content of amylose and gelatinization temperature and enhanced the starch transparency gel consistency and swelling power. In addition, biochar can improve the water solubility of starch and the gelatinization enthalpy. Importantly, the combination of biochar and nitrogen fertilizer increased the proportion of A-granules, final viscosity, starch content, and the average degree of amylopectin in polymerization. Thus, this research indicates that the combinations of biochar and nitrogen fertilizer result in the most significant improvement in the quality of starch produced from broomcorn millet.

## 1. Introduction

Broomcorn millet (*Panicum miliaceum* L.), commonly known as yellow rice, is the oldest crop in the world [1]. It has the advantages of a need for little water, a short life cycle, and p resistance to abiotic stress, which makes these plants a pioneer crop to improve marginal soil [2]. Broomcorn millet is favored because of its rich nutritional content. The dietary profiles of this food have been shown to be equivalent to those of cereals, including oats (*Avena sativa* L.), rice (*Oryza sativa* L.), and wheat (*Triticum aestivum* L.) [3], and it is rich in starch, protein, fat, dietary fiber, gluten-free ingredients, and mineral elements. Notably, broomcorn millet has 71.79–76.43% of starch, and depending on its amylose content, it can be categorized as waxy (low in amylose starch) or non-waxy (high in amylose starch) [4]. Bangar [5] has shown that the starch of broomcorn millet reveals a number of perspective functional properties that can be utilized as a substitute for commercial starch for the food and non-food industries. Therefore, an in-depth study of the properties of glutinous millet starch will promote its practical applications in the market.

Nitrogen (N) fertilizer has a pivotal role in grain yield and quality. It influences the biosynthesis of starch, which determines the quality of non-waxy and waxy broomcorn millet [6]. The level of N has a substantial effect on the surface of rice starch, which exhibits a low degree of order at the short-range scale as the level of N increases [7]. Increasing the amount of N in the soil increases the size of starch grains, overall starch content of the crop, yield, and even the quality of bread [8]. It was demonstrated that the content of amylose decreased and then increased with an increase in the application of N, and starches with a low amylose content became more digestible [9]. Studies have shown that the moderate application of N (100 and 200 kg N ha^−1^) increased the size of starch granules, and the peak viscosity, hot viscosity, and breakdown value, while the setback and pasting temperature decreased [10]. Although moderate amounts of N fertilizer can improve the quality of starch, a sufficient quantity of N fertilizer can maximize economy. Farmers frequently apply more fertilizer during the growth stage to increase grain output [11]. In modern agricultural systems, 10–30% of N fertilizer typically dissipates [12]. This poses a risk to the natural environment and its ecosystems.

A variety of natural and modified substances have been utilized as part of human efforts to preserve the environment and reduce soil pollution. Biochar (B) is one of the newly invented eco-friendly materials with an enormous surface area [13]. When N fertilizers are used with biochar, the large specific surface area of charcoal keeps the N for the plant. This boosts N efficiency and reduces leaching [14]. In addition, the addition of biochar to the soil increased the temperature, nitrogen utilization rate, and water holding capacity [15]. The application of biological carbon can effectively improve the rate of utilization of N fertilizer [16] and avoid the adverse effects of excessive application of N fertilizer on the quality of crop starch. Rapid visco analyzer (RVA) profile features, such as peak viscosity, trough viscosity, breakdown, final viscosity, and setback, were shown to be enhanced by biochar at 30 t ha^−1^ together with 135 kg N ha^−1^ [17]. The right quantity of biochar can modify the synthesis, content, and structural properties of starch by affecting its apparent quality, viscosity, and cooking quality [18]. The usage of biochar helps to mitigate the environmental issues created by the overuse of N fertilizer [14]. However, there are few reports of the impacts of biochar on starch characteristics, either on their own or when combined with N fertilizer.

To our knowledge, the effects of biochar and N fertilizer mixtures on the quality of cereal starch have rarely been studied. Consequently, the purpose of this research was to assess the impact of applying biochar, N fertilizer, and their combination on the quality of starch from two different types of broomcorn millets. The gelatinization, thermal characteristics, distribution of amylopectin chain lengths, particle size distribution, and morphology of starch were analyzed.

## 2. Materials and Methods

### 2.1. Broomcorn Millet Samples

A field experiment was conducted at the Yulin Institute of Agricultural Sciences, Yulin, China (36.17–45.60 N and 106.23–122.82 E, altitude 1229 m) from May to October 2021. Two varieties of broomcorn millet (Shanmi1-SM1 and Shanmi2-SM2), which were provided by the Small Grains Research Group of Northwest A & F University (Xianyang, China), were used in this experiment. SM1 is waxy, and SM2 is non-waxy. Three treatments of either N fertilizer (N), biochar (B), or a combination were used, including 150 kg N ha^−1^ (N150), 10 t biochar ha^−1^ (N0+B), and 150 kg N ha^−1^ plus 10 t biochar ha^−1^ (N150+B). In addition, we also used a blank control (N0) (Table 1).

### 2.2. Isolation and Purification of Broomcorn Millet Starch

Broomcorn millet grains were shelled, threshed, and ground into flour using a high-speed universal crusher (FW-100D, XinBoDe Instruments Ltd., Tianjin, China). The starch was extracted from the different treatments using the alkaline immersion method [4]. First, 100 g of broomcorn millet flour was added to 1 L of 0.1% NaOH, stirred, and stored at room temperature for 12 h. Next, this suspension was filtered consecutively through 100- and 200-mesh sieves and centrifuged at 4000 rpm for 10 min. The residue was then washed with 0.1% NaOH and centrifuged three to four times. After that, the precipitate was dissolved in distilled water, and the pH was adjusted to 7 with HCl (0.1 mol/L) following three washes with distilled water. Next, the precipitate was dried at 45 °C in an oven for 24 h. Finally, the precipitate was ground into powder and sifted through a 100-mesh sieve.

### 2.3. Morphological Observation of Broomcorn Millet Starch

The starch granules were soaked in 50% glycerin and observed with a BX53 microscope (Olympus, Tokyo, Japan) under polarized light to evaluate their micromorphology and the presence of a Maltese cross.

The starch surface structure was observed using scanning electron microscopy (Hitachi S-3400N; Hitachi, Tokyo, Japan). A thin layer of starch particles was glued to aluminum stubs with double-sided tape and sputter-coated gold. The micrographs of starch samples were taken at an accelerating voltage of 10 kV and a magnification of 3000×.

### 2.4. Granule Particle Size of Broomcorn Millet Starch

The starch particle size was measured using a laser diffraction particle size analyzer (Mastersizer 2000E; Malvern Panalytical, Westborough, MA, USA), which can analyze starch particles from 0.1 to 1000 μm [19]. Starch samples of different treatments were placed in tap water and stirred at 2000 rpm.

### 2.5. Determination of the Starch Content

The percentage of starch in the dried flour was determined using the anthrone-H_2_SO_4_ method [20]. The starch amylose content was determined by the iodine-binding method [21]. First, 10 mg of starch was weighed and placed in a 15 mL centrifuge tube followed by 1 mL of 1 mol/L NaOH and 0.1 mL of anhydrous ethanol. After the mixture was evenly mixed, it was placed in a water bath at 35 °C for 30 min. After the starch paste had been isolated, 8.9 mL of distilled water was added. Next, 200 µL of the concentrated solution was neutralized, and the pH was adjusted to 7 with HCl (0.1 mol/L). A volume of 200 µL iodine reagent and 9.4 mL of distilled water were added and thoroughly mixed. Finally, the mixture was incubated for 20 min at room temperature in the dark. The amylose content of the starch samples was obtained at 620 nm using a UV-Vis spectrophotometer (Blue Star B, Lab Tech Ltd., Beijing, China). The amylopectin contents were determined by an amylopectin test kit (Beijing Solarbio Science & Technology, Beijing, China).

### 2.6. Distribution of the Chain Length of Amylopectin

High-performance anion exchange chromatography was utilized to analyze the distribution of length of AP branch of starch [22]. Flow rate, 0.4 mL/min; injection volume, 5 μL; solvent system, 0.2 M NaOH (0.2 M NaOH, 0.2 M Na acetate); gradient program, 90:10 *v*/*v* at 0 min, 90:10 *v*/*v* at 10 min, 40:60 *v*/*v* at 30 min, 40:60 *v*/*v* at 50 min, 90:10 *v*/*v* at 50.1 min, and 90:10 *v*/*v* at 60 min.

### 2.7. Structure of Broomcorn Millet Starch

The ordered structure of the starch outer region was analyzed using attenuated total reflectance–Fourier transform infrared (ATR–FTIR) spectroscopy (7000 Varian; Agilent Technologies, Santa Clara, CA, USA).

### 2.8. Solubility and Swelling

The swelling power and water solubility of the starch from broomcorn millet were determined at 60, 70, 80, and 90 °C. A total of 200 mg of the starch sample was weighed into a dry 15 mL centrifuge tube. A volume of 10 mL of distilled water was added and mixed thoroughly. Each tube was incubated in a water bath at 60, 70, 80, and 90 °C for 30 min, removed, and cooled to room temperature. Each tube was then centrifuged at 2000 rpm for 20 min. The supernatant was immediately poured into an aluminum box, which was cleaned, dried, and weighed, and the remaining substance was weighed in the test tube. The amount of starch that dissolved is considered to be the residue of the supernatant after drying, and the swelling power of starch was calculated from the remaining amount in the test tube.

An aqueous dispersion of 1% starch was placed in a water bath at 100 °C for 20 min and then cooled to room temperature. The light transmittance of starch was then measured using a UV-Vis spectrophotometer (Lab Tech Ltd., Beijing, China) at 620 nm.

### 2.9. Determination of Gel Consistency and Pasting Properties

The gel consistency of starch was determined as described by Chemutai et al. [23]. Briefly, the starch was stained using thymol blue. Potassium hydroxide (KOH) (0.2 N) was added, and the sample was mixed well. Glass marbles protected the tubes from steam and sample reflow. The sample was boiled in a 92 °C water bath for 6 min, incubated at room temperature for 5 min, and then on ice for 15 min. Finally, the samples were placed horizontally on millimeter square paper, and the results were observed after 1 h.

The starch regeneration rate was determined as described by Chao et al. [24] with modifications. Starch paste (1%, 15 mL) was prepared, placed in a scaled test tube, and incubated at room temperature for 48 h. Finally, the volume of supernatant in each test tube was measured every 2 h. The coagulation curve was based on the percentage of supernatant in the total volume of the pastes over time.

Broomcorn millet starch (3.0 g) was weighed and mixed with 25 mL distilled water in the sample container of a rapid viscosity analyzer (RVA 4500; Perten Instruments AB, Stockholm, Sweden). The test cycle was as follows: the sample was heated to 50 °C, held for 1 min, heated to 95 °C at 12 °C/min, maintained at 90 °C for 2 min, and cooled to 50 °C at 12 °C/min. The peak viscosity (PV), breakdown viscosity (BV), final viscosity (FV), setback viscosity (SV), and pasting temperature (PT) were obtained.

### 2.10. Thermal Properties

Differential scanning calorimetry (DSC; Q2000, TA Instruments, Wood Dale, IL, USA) was used to determine the thermal properties of the broomcorn millet starch. First, starch (3.0 mg) was immersed in ultrapure water (9.0 μL) in an aluminum pot, which was sealed. The prepared samples were then balanced at room temperature for 4 h. The DSC then heated the sample from 40 °C to 120 °C at 10 °C/min. A sealed empty aluminum pot was used as the control.

### 2.11. Statistical Analysis

All the data were obtained in three biological replicates. The data in this study were presented in the form of means ± SD. In addition, one-way and two-way analyses of variance (ANOVA) by a Tukey’s test were evaluated using SPSS 19.0 (IBM, Inc., Armonk, NY, USA) and Origin 2021 for drawing (OriginLab, Northampton, MA, USA). Data of the distribution of the chain length of amylopectin were acquired on an ICS 5000 Dionex HPLC (Thermo Fisher Scientific, Waltham, MA, USA), and processed using Chromeleon 7.2 CDS (Thermo Fisher Scientific). The quantified data were outputted into Microsoft Excel 2021 (Redmond, WA, USA).

## 3. Results

### 3.1. Scanning Electron Microscopy (SEM) of Starch

**Figure 1:**
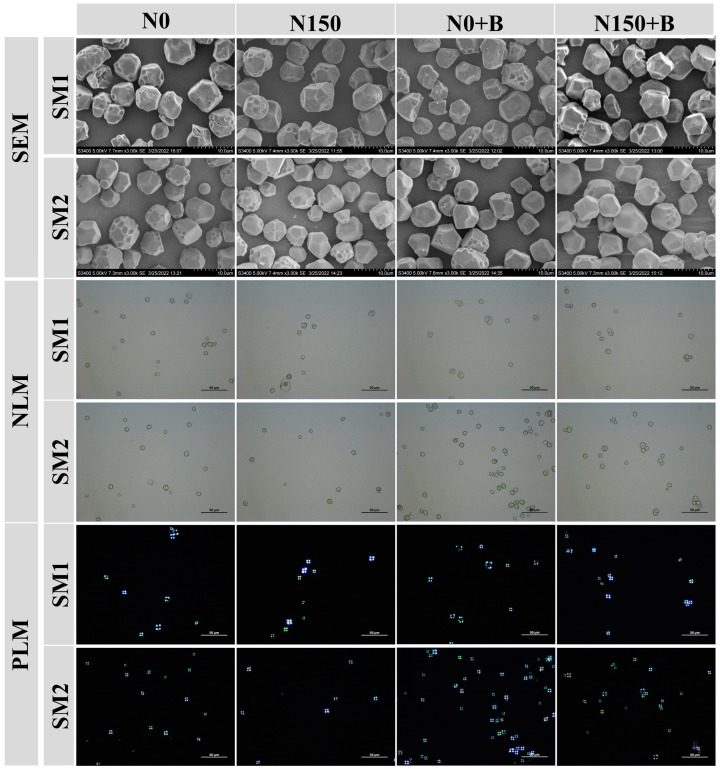
The morphologies of starch granules under normal light microscopy (NLM), polarized light microscopy (PLM), and scanning electron microscopy (SEM). N0, control; N150, nitrogen fertilizer; N0+B, biochar; N150+B, nitrogen fertilizer and biochar.

Starches obtained from different treatments were spherical and showed regular polygons and the typical Maltese cross (Figure 1). It is worth noting that most starches have pits on their surfaces, presumably because of alkali corrosion during extraction. The surface of fertilizer-treated starch grains was found to be smoother compared with N0, particularly, N150+B. In addition, the starch granules had a more diverse morphology in SM2 compared with SM1.

### 3.2. Distribution of Starch Granule Sizes

**Figure 2:**
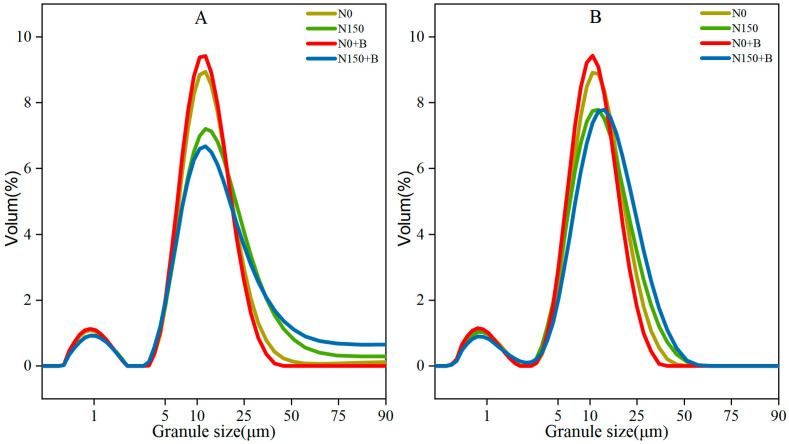
The distribution of starch granule sizes. (**A**) SM1; (**B**) SM2. N0, control; N150, nitrogen fertilizer; N0+B, biochar; N150+B, nitrogen fertilizer and biochar.

The volume of broomcorn millet starch under eight treatments showed a bimodal distribution (Figure 2A,B). The granule size distribution was classified as A-granules (>15 μm), B-granules (5–15 μm), and C-granules (<5 μm) [25]. In SM1 and SM2, the B-granules contributed the most to total volume with an average of 53.90% and 57.71%, respectively (Table 2). N0+B decreased the proportion of A-granules, while increasing the proportions of B-granules and C-granules compared with N0, whereas N150 had the opposite effect. Intriguingly, N150+B exhibited the same trends of action as N fertilizer but with a greater impact. In particular, there was a 50.76% and 52.33% increase in N150+B compared with N0 in SM1 and SM2, respectively. In addition, there was a consistent trend in the effect of each treatment between varieties. A significant variation in the distribution of starch grain sizes was observed among the different millet varieties, all the treatments, and their interactions, except for the C-granules (Table 2), indicating that both the variety of millets and their individual treatments play a role in influencing the distribution of starch grain size.

### 3.3. Starch Content

Table 3 demonstrates that the total starch content differed significantly between the treatments. In this study, the N150 treatment significantly increased the total starch content compared with N0. However, N150+B further increased the total starch content. This is consistent with previous research [26], which reported that the application of N significantly increased the starch content of sweet potato (*Ipomoea batatas* [L.] Lam.). N150+B did not have any notable impact on SM1, whereas it significantly elevated the overall starch content in SM2 compared with N0, which resulted in a 3.19% increase. An ANOVA suggested that the treatments and varieties had a significant influence on the total starch. The amylose contents of waxy and non-waxy broomcorn millet ranged from 2.97% to 4.00% and 28.65% to 33.18%, respectively. However, there were no apparent significant differences among the various N and biochar treatments of SM1 (Table 3). The findings indicated that the various interventions led to a decrease in the level of linear starch present in SM2, particularly, N150+B by 13.65%. There was a decrease observed in the levels of amylose in the N150+B treatment in SM1, although it was not statistically significant. All the treatments increased the amylopectin content of non-waxy broomcorn millet, but only the effect of N150+B was significant (Table 3). However, there was no significant difference among the treatments in SM1. The starch consisted of both amylose and amylopectin.

### 3.4. Distribution of the Chain Lengths of Amylopectin

**Figure 3:**
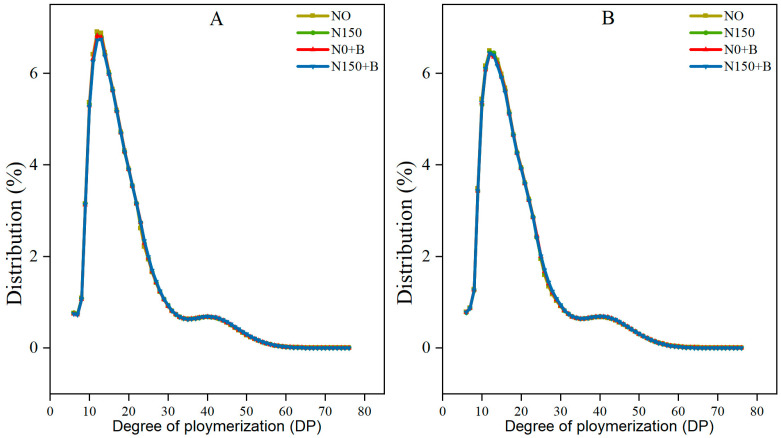
The distributions of the AP chain length of amylopectin. (**A**) SM1; (**B**) SM2. N0, control; N150, nitrogen fertilizer; N0+B, biochar; N150+B, nitrogen fertilizer and biochar.

The distribution of amylopectin (AP) chain lengths significantly influenced the pasting properties, gel properties, and crystalline structure of the starches [27]. The distributions of AP chain length are shown in Figure 3A,B. The starch of all the treatments showed a bimodal distribution, and the main peaks were slightly different and occurred at the level of depolymerization (DP) 12–13. The amylopectin branch chains may be broken down into the following categories: DP 6–12 (A chain), DP 13–24 (B1 chain), DP 25–36 (B2 chain), and DP ≥ 37 (B3+chains). Table 4 shows that the AP of each treatment had the highest percentage of B1chains. The treatments resulted in an increase in the average length of polymer chains with SM1 showing a higher degree of polymerization compared with its counterpart in SM2. Furthermore, the treatment N150+B exhibited a pronounced impact by enhancing the average degree of polymerization (%), particularly in SM2 where it displayed a notable distinction from the remaining treatments. Fertilizer treatments increased the ratio of B3+chains compared with those of N0, and the N150+B treatment had the most significant effect. The percentage of B3+chains was generally higher in waxy than in non-waxy broomcorn millet. The results of a two-way ANOVA indicated that Varieties and Treatments had a significant impact on the distribution of chain lengths in all the chains except for B2. Additionally, there was only a significant interaction between Varieties and Treatments in the A1 and B1 chains. We found that fertilizer reduced the proportion of short chains in AP and increased the gelatinization onset (To), peak temperature (Tp), and gelatinization enthalpy (ΔH).

### 3.5. ATR–FTIR Analysis of Starch

**Figure 4:**
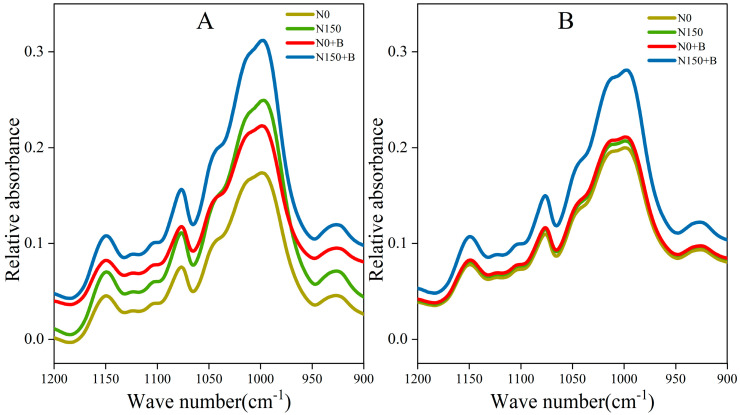
Ordered structure (FTIR) of starch. (**A**) SM1; (**B**) SM2. FTIR, Fourier transfer infrared spectroscopy; N0, control; N150, nitrogen fertilizer; N0+B, biochar; N150+B, nitrogen fertilizer and biochar.

The short-range ordered structure of broomcorn millet starch can be reflected by ATR–FTIR spectroscopy. The ATR–FTIR spectra of waxy and non-waxy broomcorn millet starch after different treatments are shown in Figure 4A,B. The most significant changes were found in the 900–1200 cm^−1^ region. The degree of order was quantified by the ratio of 1045/1022 cm^−1^, and the ratio of amorphous to ordered carbohydrate structure in the starch was 1022/995 cm^−1^ [28]. There were consistent main characteristic peaks of broomcorn millet starch under different treatments. In SM1, the infrared (IR) ratio of 1045/1022 cm^−1^ ranged from 0.687 to 0.761, and N150+B of SM1 had the highest peak (Table 5). Our results indicated that the 1045/1022 values were elevated, while the 1022/995 values were reduced across all the interventions. The 1045/1022 value for SM2 exceeded that of SM1, while the inverse was true for the 1022/995 value. The outcomes of the two-factor ANOVA indicated that both 1045/1022 and 1022/995 were significantly influenced by treatment and variety.

### 3.6. Swelling Power and the Water Solubility of Starch

**Figure 5:**
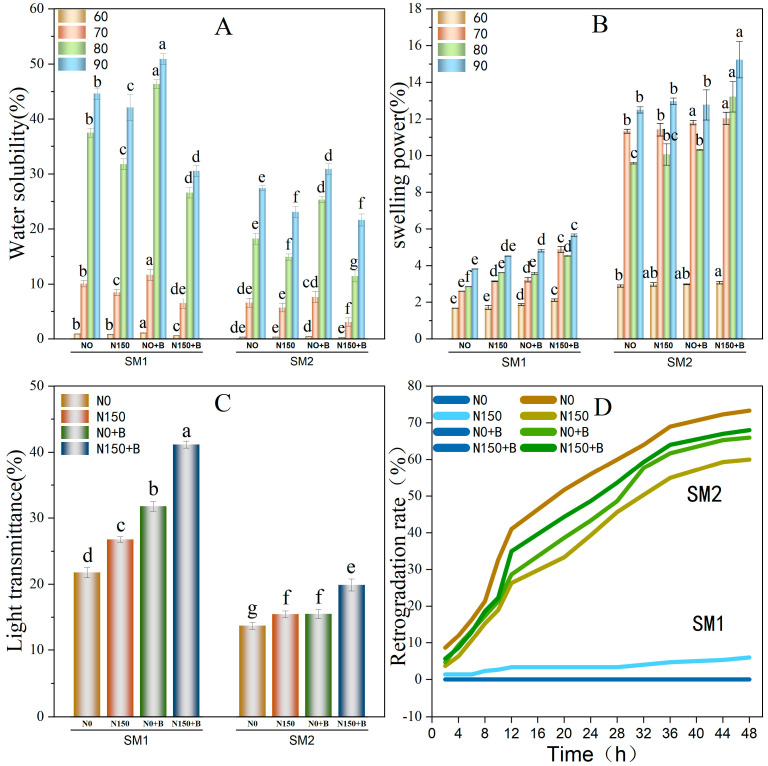
(**A**) Water solubility of starch; (**B**) Swelling power of starch; (**C**) Light transmittance of starch; (**D**) Retrogradation curves of starch. Different letters indicate variability among the eight different treatments. N0, control; N150, nitrogen fertilizer; N0+B, biochar; N150+B, nitrogen fertilizer and biochar.

Figure 5A summarizes the water solubility of the two varieties under various treatments at different temperatures (60 °C, 70 °C, 80 °C, and 90 °C). The temperature substantially influences the water solubility of broomcorn starch, and the swelling capacity and water solubility of broomcorn millet starch increase in parallel with the temperature. Higher temperatures increased the mobility of molecular chain, which increased the distribution of sugar molecules, and thereby improved solubility. The waxy broomcorn millet was more soluble compared with the non-waxy broomcorn millet. The solubility of broomcorn millet starch in water was significantly reduced by N150 compared with N0. In contrast, N0+B significantly increased the solubility of starch in water, while N150+B decreased this parameter.

Water solubility refers to the solubility of starch during the process of swelling, while the expansion potential refers to the water holding capacity of starch after heating, cooling, and centrifugation [29]. The starch expansion potential barely changed at 60 °C and was relatively stable (Figure 5B). Compared with N0, the swelling degree of starch in the N150+B treatment increased, while it decreased in N0+B. The potential of the starch to expand also increased in N150. Notwithstanding, there was a relatively high potential for expansion in the N150+B treatment.

### 3.7. Light Transmittance of Starch

Transmittance describes the degree of transparency of starch paste. There were significant differences in light transmittance between waxy and non-waxy broomcorn millet starch under different treatments. The light transmittance of the waxy broomcorn millet starch paste varied between 21.75% and 41.15%, while the non-waxy variety ranged between 13.71% and 19.87% (Figure 5C). Our results showed that the light transmittance of broomcorn millet starch increased following the application of either N0+B or N150. In addition, N150+B further increased the light transmittance. This synergic effect may be owing to the increased proportion of A-granules in the starch combined with the decrease in amylose content.

### 3.8. Retrogradation

The coagulation curves of broomcorn millet starch under different biochar and N fertilizer treatments are shown in Figure 5D. There were significantly different degrees of retrogradation between the two varieties. In SM2, the percentages of retrogradation at 48 h were 60.00–73.33%. There were consistent changes in trends in the percentages of paste retrogradation in the four SM2 treatments. The retrogradation percentages of N150 were lower than those of N0, and the retrogradation percentages of N0+B were lower than those of N150+B. Thus, the application of N fertilizer alone attenuated the starch retrogradation. Alternatively, N0+B and N150+B reduced the starch retrogradation percentages compared with N0.

### 3.9. Gel Consistency of Starch

The length of gel ranged from 8.01 mm to 12.24 mm in SM1 and from 5.74 mm to 6.36 mm in SM2 (Table 5), respectively. However, the gel consistency of SM1 was more sensitive to fertilizer disturbance compared with that of SM2. In this study, amendment with fertilizer significantly reduced the content of amylose and pasting temperature and increased the gel consistency of the starch. These findings indicate that fertilizer treatments have a discernible positive impact on the cooking quality of broomcorn millet.

### 3.10. Pasting Properties

N fertilizer and biochar significantly affected the gelatinization properties of broomcorn millet starch (Table 6). The PV represents the maximum viscosity of starch when heated in water. The highest start of PV in SM2 was in N150+B. In addition, the proportion of A-granules and the swelling potential of broomcorn millet starch were higher under this treatment. Thus, it is possible that the accelerated rate of water uptake by the starch granules ultimately increased their PV. We observed a significant disparity in the PV between SM1 and SM2 with SM1 exhibiting notably higher levels. Additionally, the introduction of N150 demonstrated a substantial enhancement in PV, particularly within SM1, whereas no significant impact was observed in SM2. This may be related to the difference in genotype, amylose content, and molecular weight. In SM1, the starch BV after the N150 and N150+B treatments was higher than that of the N0, while the starch BV after the N150+B treatment was significantly lower. This shows that the application of N reduces the heat resistance of millet starch, and the combination of biochar and N can significantly improve this parameter. In contrast, the N150 and N0+B treatments reduce the BV of broomcorn millet starch in SM2. The disparity could possibly be owing to differences in the genotypes and varieties. Furthermore, the statistical significance (*p* < 0.05) of both the individual and combined impacts of biochar and N fertilizer, as well as the influence of different varieties, were observed. The SV indicates the performance of the starch paste after cooling. A higher value indicates a greater tendency to recede. In SM1, the SV was the highest under the N150 treatment and was significantly different from that of the other treatments. In SM2, the SV ranged from 394 to 648 mPa s and was the highest in the N150+B treatment. This is consistent with our retrogradation results. Compared with the non-waxy broomcorn millet, the waxy variety has a lower SV, which could explain the difference between varieties. The highest PT was observed for N0 in SM2 across all the treatments, and it consistently showed higher values in SM2 compared with those of SM1. The application of N fertilizer and biochar in SM1 resulted in decreased PT values compared with N0, although no significant variation was observed among the different treatments. The parameter denoted as FV refers to the viscosity of starch paste following a 50 °C holding period at the conclusion of the RVA test [30]. A statistical analysis revealed that the pasting characteristics of broomcorn millet starch were significantly influenced by treatment, variety, and their interaction. However, it was observed that the FV remained unaffected by the variety factor.

### 3.11. Differential Scanning Calorimetry (DSC)

The thermal properties of broomcorn millet starch samples under different treatments were measured by differential scanning calorimetry (DSC) (Table 7). In SM1, N150 significantly increased the ΔH of starch. In SM2, the increase in ΔH was not statistically significant. However, the starch values of Tp, To, and ∆H were higher in SM1 than in SM2. In addition, both N150 and N150+B significantly increased the To in SM1. We observed a notable disparity between SM1 and SM2 in the values for Tp, and an ANOVA showed that the different varieties significantly impacted Tp. In SM1, the highest Tp values were found in N0+B, which may be related to the proportion of B1 chains.

## 4. Discussion

In this study, most starch grains exhibited irregular polygonal shapes, and the N150+B treatment resulted in smoother grains compared with those of the other treatments. Starch grain morphology is influenced by a variety of factors, including amylose content, and starch with a higher amylose content has irregular grains [31]. Previous studies have shown that a lower amylose content will probably result in smoother starch grains [32]. Based on the results of this study, compared with the control, each treatment significantly reduced the amylose content. In addition, both N150 and N150+B increased the percentage of A-granules, particularly, those of N150+B. We hypothesized that the application of biochar can strengthen the effect of N applications on the size of broomcorn millet starch granules. The application of relevant N fertilizer can enhance the process of grain-filling and starch synthesis. The A-granules usually develop at an early stage [33], which could be the reason why N150+B improves the growth of broomcorn millet endosperm at an early stage of development. Moreover, biochar improved the bioavailability of N for growth and development, which has been confirmed in rice [34]. The silicon contained in biochar is bioavailable to plants, and some studies have reported that silicon fertilizer can improve the appearance and starch content of seeds [35]. The dissimilarities observed in the distribution of particle sizes between the two cultivars could be attributed to disparities in the genotypes of varieties or the interactions between varieties and treatments. We also found that biochar increased the total starch content. It is notable that N0+B acts more significantly in SM2 compared with SM1, which may be caused by different crop genotypes. The findings of this research indicate that the total starch was significantly influenced by both variety and treatment, while no significant interaction between variety and treatment was observed in relation to the total starch. The ratio of straight chain to branched-chain starch may be influenced by the activity of starch-branching enzymes (SBE) and soluble starch synthase (SSS) that participate in the synthesis of branched-chain starch in grains [36]. Zhang et al. [37] concluded that the application of N fertilizer led to a reduction in the amylose content. The results of this study may be attributed to the fact that the application of biochar enhanced the effect of N fertilizer and increased the activities of SBE and SSS, which may be responsible for the decrease in amylose content [38]. A notable dissimilarity was observed in amylose content between the two cultivars, and there was a remarkable interplay between treatment and cultivar on amylose content. The application of biochar has been shown to reduce amylose content in rice [18]. Amylose content is important in digestion because starch with a low amylose concentration is easier to digest and absorb [39]. Therefore, broomcorn millet starch under N150+B is a suitable raw material to produce food suitable for children, the elderly, and people with weak digestive function. The reduction in amylose content increases the tensile strength of flour [39]. Thus, the application of biochar and N fertilizer is crucial to enhance the quality of broomcorn millet starch. Li et al. [40] reported that starches that have greater proportions of long AP chains (DP ≥ 37) were more effective at swelling. Consistent results were also observed in this study. The administration of N150+B resulted in a significant increase in the percentage of DP ≥ 37 for both varieties. Furthermore, a significant effect was observed for both variety and treatment individually (*p* < 0.01), but there was no detectable significant interaction between the two factors. Some studies have shown that the distribution of AP chain length can influence its pasting properties. We found that fertilizer reduced the proportion of short chains in AP and increased the To, Tp, and ∆H. This may be because a decrease in short chains in AP increases the packaging efficacy within the crystal structure of starch, which leads to a higher To, Tp, and ∆H [41].

The water solubility of starch can be inhibited by its amylose content [42], which inhibits the destruction of the amylopectin double helix structure. This also explains why waxy broomcorn millet is significantly more soluble than non-waxy broomcorn millet. We found that the solubility of starch was significantly decreased by N150 and N150+B and increased by N0+B. This may be because the small granules are more hydrophilic than the large ones [43]. This was also confirmed by the results of the distribution on granule size in this study. The solubility of starch in water is proportional to its swelling degree. SM1 transmits more light than SM2 and transmitted more N150+B across all the treatments. The utilization of a combination of biochar, chemical, and organic nitrogen in the proportion of 80:10:10 led to a notable enhancement in the light transmittance of starch [44]. The grain size, amylose content, and amylose/amylopectin ratio of starch all affect the light transmittance of starch paste [6]. A higher content of large particles in the starch paste results in more light transmittance [45]. Light transmittance is one of the important indices of starch paste, and starch products that are highly transparent will taste better. Clearly, compared with other treatments, the N150+B broomcorn millet starch will be strongly preferred to other types of starch from this crop.

The retrogradation percentages of the starch paste of waxy broomcorn millet were significantly lower than those of non-waxy millet, which may be related to the differences in genotypes between these types of broomcorn millet [46]. The application of N fertilizer alone attenuated starch retrogradation. Similar results were obtained for common buckwheat (*Fagopyrum esculentum* Moench) [47]. The retrogradation of starch paste is a significant factor that limits the application of starch, which affects the mechanical properties of starch food and changes its nutritional value and taste. In addition, the retrogradation percentages of starch are affected by grain structure, gelatinization temperature, and moisture content [48]. Because of its high transparency and low retrogradation percentages, broomcorn millet starch has the potential to be developed as a resource to prepare beverages [5]. N150+B increases the transparency of starch and reduces its precipitation, thus, providing a basis for millet starch as a raw material for beverages. The amylose level, gel consistency, and gel temperature are the three primary physicochemical parameters that may be used to characterize eating and cooking quality [49]. Cao et al. [50] reported that the variation in amylose content led to the variation in gel consistency. Starch is composed of both helical structures that are connected by hydrogen bonds to create a strong crystalline structure, as well as dispersed regions within the amorphous area of the molecule. Previous research has indicated that the application of nitrogen-based fertilizer elevates the level of short-range ordered configuration in starch, a finding that is consistent with those of our study [10]. Furthermore, our findings indicate that the addition of N150+B to SM1 leads to a significant increase in the 1045/1022 value and decrease in the 1022/995 value compared with N0, which suggests an enhancement of the short-range ordered structure of SM1.

The starch pasting temperature was reduced for both varieties of broomcorn millet whether biochar was applied alone or in combination with biochar and N fertilizer, and these results were statistically significant (*p* < 0.05). This increases the ease of gelatinizing the treated starch. Interestingly, various varieties of rice behaved differently when treated with biochar (10 t hm^−2^) [18]. Treatment with biochar reduced the pasting temperature of the starch from the variety Akihikari, while treatment with biochar had no such effect on the SN265 variety. This difference could probably be attributed to the differences between the distribution of starch granules, the content of amylose, and the proportion of amylose and amylopectin [51]. The BV represents the heat resistance of starch, and a lower BV within a specific range results in higher heat resistance [52]. In this study, N150+B significantly reduced the BV of starch, indicating that N150+B can increase its heat resistance. Gao et al. [53] studied the application of N fertilizer to buckwheat and found that it can improve the heat resistance of its starch. PV reflects the expansion of starch granules and is the maximal viscosity of gelatinized starch when heated in water [54]. We found that N150+B in SM2 had the highest PV of starch. After the treatment was administered, there was a noticeable increase in PV. This indicates that biochar or N fertilizer may enhance the rate of swelling of broomcorn millet starch granules, which causes them to quickly absorb water and ultimately increase their PV. In addition, the proportion of A-granules and the swelling potential of broomcorn millet starch were higher under this treatment. Many factors affect the thermal properties of starch, including the shape and size of granules, the distribution of AP chain length, and the influence of crystal structure [55]. In SM2, N150+B had the lowest gelatinization temperature and ∆H, probably owing to the lowest percentage of amylose and the highest percentage of A-granules. A high amylose content will increase the gelatinization temperature of starch owing to blockage of the gelatinization of crystallinity [56]. The PT refers to the temperature at which the viscosity begins to increase owing to heating, and it represents the minimum threshold for this phenomenon [57]. The dissimilarity in PT between the two varieties can be accounted for by the variety, which had a statistically significant impact on PT (*p* < 0.05). Wang et al. [58] showed that a high proportion of B1 chains favors the dominant accumulation of amylopectin chains in the crystalline regions, which renders these structures thermally resistant. The gelatinization temperature of starches is crucial for selecting target varieties with adequate physicochemical properties to meet all the requirements for the applications of food and non-food.

**Figure 6:**
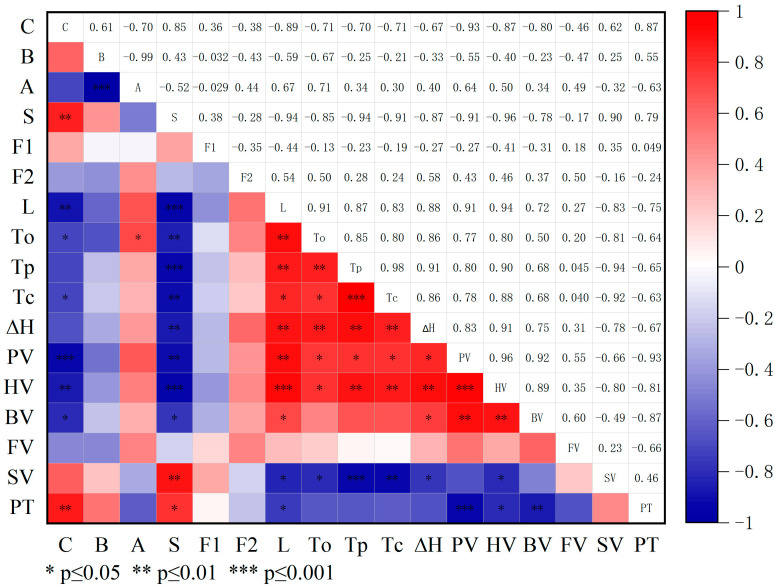
Pearson’s correlation coefficients of the physicochemical properties on broomcorn millet starch. A-granules (>15 μm), B-granules (5–15 μm), and C-granules (<5 μm); S, amylose content; F1, 1045/1022; F2, 1022/955; L, Light transmittance; To, onset gelatinization temperature; Tp, peak gelatinization temperature; Tc, conclusion gelatinization temperature; ΔH, gelatinization enthalpy; PV, peak viscosity; HV, hot viscosity; BV, breakdown viscosity; FV, final viscosity; SV, setback viscosity PT, peak viscosity. Data are the means of three replicates.

A Pearson correlation analysis was utilized to analyze the physicochemical properties on broomcorn millet starch, and the results are shown in Figure 6. In particular, the transparency of the starch paste negatively correlated with the C-granules and B-granules but positively correlated with the A-granules. Starch pastes with a higher proportion of large particles are reported to contain less granular residue, which enables the passage of light; this results in higher light transmission [44]. In addition, the amylose content positively correlated with the C-granules and B-granules. This may be owing to the combined application of biochar and N fertilizer, which can inhibit the conversion of large particles into small particles. In contrast, Sing et al. [59] showed that the content of amylose was not related to the proportion of A-, B-, and C-granules. This may vary depending on the type of crop.

## 5. Conclusions

These results showed that biochar and N fertilizer increase the smoothness of the surface of starch particles and change their particle size distribution. For both varieties, the proportion of A-granules increased significantly in the N150+B treatment, while the proportion of B-granules and C-granules decreased. Importantly, N150+B has the lowest amylose content and the best light transmittance. The starch after N treatment has the lowest trend of retrogradation in SM2. Biochar can increase the water solubility of millet and needs more energy for the gelatinization of broomcorn millet. The heat resistance of SM1 was enhanced by N150+B. The addition of biochar can increase the thermal stability of broomcorn millet starch and improve its gelatinization characteristics. In summary, the utilization of biochar has the potential to augment the efficacy of N fertilizer, elevate the levels of seed starch, and enhance the characteristics of starch.

## Figures and Tables

**Table 1:** The meanings and abbreviations in all the tables.

Varieties	Treatments	Specific Measures
SM1 (Shanmi1)	N0	No treatment
	N150	Nitrogen fertilizer (150 kg N ha^−1^)
	N0+B	Biochar (10 t hm^−2^)
	N150+B	Nitrogen fertilizer and biochar (150 kg N ha^−1^ and 10 t hm^−2^)
SM2 (Shanmi2)	N0	No treatment
	N150	Nitrogen fertilizer (150 kg N ha^−1^)
	N0+B	Biochar (10 t hm^−2^)
	N150+B	Nitrogen fertilizer and biochar (150 kg N ha^−1^ and 10 t hm^−2^)

**Table 2:** The impact of distinct interventions on the distribution of starch particles and the application of an analysis of variance (ANOVA) to evaluate the individual treatments and varietal interactions across various ranges of particle sizes.

Varieties	Treatments	Distribution of Starch Granules (%)		
		C (<5 μm)	B (5–15 μm)	A (>15 μm)
SM1	N0	9.55 ± 0.17 de	58.73 ± 1.54 b	31.72 ± 1.70 c
	N150	9.15 ± 0.32 e	49.86 ± 2.74 c	40.99 ± 3.07 b
	N0+B	10.16 ± 0.34 c	63.41 ± 2.40 a	26.43 ± 2.73 d
	N150+B	8.61 ± 0.30 f	43.57 ± 1.78 d	47.82 ± 2.07 a
Average		9.37%	53.90%	36.74%
SM2	N0	10.79 ± 0.17 b	61.31 ± 1.23 ab	27.90 ± 1.40 cd
	N150	11.20 ± 0.41 ab	58.19 ± 2.98 b	30.61 ± 2.61 c
	N0+B	11.39 ± 0.23 a	63.70 ± 1.50 a	24.91 ± 1.73 d
	N150+B	9.86 ± 0.21 cd	47.64 ± 1.88 c	42.50 ± 2.08 b
Average		10.81%	57.71%	31.48%
Analysis of Variance	Varieties	**	**	**
	Treatments	**	**	**
	Varieties × Treatments	ns	*	*

Note: Different letters within a column indicate significant difference among the mean values (*p* < 0.05). Data are the means of three replicates. N0, control; N150, nitrogen fertilizer; N0+B, biochar; N150+B, nitrogen fertilizer and biochar. ns, nonsignificant. * *p* < 0.05. ** *p* < 0.01.

**Table 3:** Total starch, amylose, and amylopectin contents of broomcorn millet starch under different treatments and the application of an analysis of variance (ANOVA) to evaluate the individual treatments and varietal interactions on the contents of starch.

Varieties	Treatments	Total Starch Content (%)	Amylose Content (%)	Amylopectin Content (%)
SM1	N0	62.39 ± 1.41 e	4.00 ± 1.33 c	96.00 ± 1.33 a
	N150	65.22 ± 0.82 d	3.48 ± 0.83 c	96.52 ± 0.83 a
	N0+B	63.12 ± 0.68 e	3.37 ± 1.00 c	96.63 ± 1.00 a
	N150+B	66.53 ± 2.19 d	2.97 ± 0.70 c	97.03 ± 0.70 a
SM2	N0	69.18 ± 0.59 c	33.18 ± 2.03 a	66.82 ± 2.03 c
	N150	71.13 ± 0.68 b	30.51 ± 1.26 b	69.49 ± 1.26 b
	N0+B	71.39 ± 0.57 ab	30.47 ± 0.62 b	69.53 ± 0.62 b
	N150+B	73.19 ± 0.97 a	28.65 ± 1.79 b	71.35 ± 1.79 b
Analysis of Variance	Varieties	**	**	**
	Treatments	**	**	**
	Varieties × Treatments	ns	*	ns

Note: Different letters within a column indicate significant differences among the mean values (p < 0.05). Data are the means of three replicates. N0, control; N150, nitrogen fertilizer; N0+B, biochar; N150+B, nitrogen fertilizer and biochar. ns, nonsignificant. * *p* < 0.05. ** *p* < 0.01.

**Table 4:** Distribution of chain length and the average degree of polymerization of amylopectin. An analysis of variance (ANOVA) was utilized to assess the effect of treatment and variety interactions on the properties of starch.

Varieties	Treatments	Chain Length Distribution (%)				Average Degree of Polymerization (%)
		DP 6–12	DP 13–24	DP 25–36	DP ≥ 37	
SM1	N0	24.50 ± 0.20 a	54.51 ± 0.12 b	12.06 ± 0.14 b	8.93 ± 0.18 bc	19.55 ± 0.08 b
	N150	24.18 ± 0.08 c	54.32 ± 0.09 c	12.33 ± 0.11 ab	9.17 ± 0.06 b	19.66 ± 0.04 a
	N0+B	24.21 ± 0.06 bc	54.15 ± 0.04 d	12.47 ± 0.04 a	9.18 ± 0.06 b	19.68 ± 0.03 a
	N150+B	24.23 ± 0.04 bc	53.92 ± 0.01 e	12.28 ± 0.38 ab	9.57 ± 0.33 a	19.71 ± 0.01 a
SM2	N0	24.39 ± 0.05 ab	54.64 ± 0.02 a	12.29 ± 0.02 ab	8.68 ± 0.05 c	19.44 ± 0.02 c
	N150	24.23 ± 0.06 bc	54.54 ± 0.07 abc	12.33 ± 0.05 ab	8.90 ± 0.08 bc	19.54 ± 0.04 b
	N0+B	24.10 ± 0.10 c	54.57 ± 0.03 ab	12.40 ± 0.04 a	8.93 ± 0.09 bc	19.57 ± 0.04 b
	N150+B	23.89 ± 0.11 d	54.44 ± 0.04 c	12.52 ± 0.04 a	9.14 ± 0.11 b	19.67 ± 0.05 a
Analysis of Variance	Varieties	*	**	ns	**	**
	Treatments	**	**	ns	**	**
	Varieties × Treatments	*	**	ns	ns	ns

Note: Different letters within a column indicate a significant difference among the mean values (*p* < 0.05). Data are means of three replicates. N0, control; N150, nitrogen fertilizer; N0+B, biochar; N150+B, nitrogen fertilizer and biochar. ns, nonsignificant. * *p* < 0.05. ** *p* < 0.01.

**Table 5:** Gel consistency and FT-IR ratios of broomcorn millet starch under different treatments. An analysis of variance (ANOVA) was utilized to assess the effect of treatment and variety interactions on the properties of starch.

Varieties	Treatments	Gel Consistency (mm)	FT-IR Ratios	
			1045/1022 (cm^−1^) (R1)	1022/995 (cm^−1^) (R2)
SM1	N0	8.01 ± 0.17 c	0.687 ± 0.001 d	0.866 ± 0.019 ab
	N150	10.63 ± 0.12 b	0.691 ± 0.002 d	0.803 ± 0.024 c
	N0+B	11.28 ± 0.54 b	0.752 ± 0.006 ab	0.857 ± 0.013 b
	N150+B	12.24 ± 0.82 a	0.761 ± 0.012 a	0.790 ± 0.053 c
SM2	N0	5.74 ± 0.22 d	0.739 ± 0.010 c	0.902 ± 0.007 a
	N150	5.86 ± 0.08 d	0.737 ± 0.008 c	0.892 ± 0.014 ab
	N0+B	6.02 ± 0.29 d	0.740 ± 0.005 bc	0.900 ± 0.010 ab
	N150+B	6.36 ± 0.43 d	0.741 ± 0.002 bc	0.886 ± 0.008 ab
Analysis of Variance	Varieties	**	**	**
	Treatments	**	**	**
	Varieties × Treatments	**	**	ns

Note: Different letters within a column indicate significant differences among the mean values (*p* < 0.05). Data are the means ± SD of three replicates. FT-IR, Fourier transfer infrared spectroscopy; N0, control; N150, nitrogen fertilizer; N0+B, biochar; N150+B, nitrogen fertilizer and biochar; ns, nonsignificant. ** *p* < 0.01.

**Table 6:** Pasting properties of starch. An analysis of variance (ANOVA) was utilized to assess the effect of treatment and variety interactions on the pasting properties of starch.

Varieties	Treatments	PV (mPa s)	BV (mPa s)	FV (mPa s)	SV (mPa s)	PT (°C)
SM1	N0	1656.67 ± 127.26 c	901.67 ± 41.65 b	841.67 ± 48.56 c	124.00 ± 20.07 d	79.17 ± 0.03 cd
	N150	2101.33 ± 157.16 a	1084.00 ± 73.18 a	1189.67 ± 19.55 a	207.67 ± 9.07 c	78.38 ± 0.06 e
	N0+B	1871.67 ± 47.54 b	1106.00 ± 101.30 a	1024.67 ± 43.00 b	127.00 ± 16.70 d	78.38 ± 0.03 e
	N150+B	1879.33 ± 61.85 b	767.67 ± 34.93 c	1021.33 ± 82.40 b	168.00 ± 11.53 cd	78.13 ± 0.46 e
SM2	N0	970.67 ± 7.64 d	502.33 ± 6.51 d	916.00 ± 4.00 bc	447.67 ± 9.01 b	80.22 ± 0.51 a
	N150	942.33 ± 15.14 d	443.67 ± 3.05 d	893.33 ± 11.23 bc	394.67 ± 9.29 b	79.48 ± 0.46 bc
	N0+B	907.67 ± 29.26 d	468.67 ± 14.98 d	912.00 ± 34.65 bc	473.00 ± 20.07 b	79.97 ± 0.02 ab
	N150+B	1496.67 ± 153.64 c	913.67 ± 91.28 b	1231.67 ± 178.40 a	648.67 ± 116.33 a	78.62 ± 0.55 de
Analysis of variance	Varieties	**	**	ns	**	**
	Treatments	**	**	**	**	**
	Varieties × Treatments	**	**	**	**	**

Note: Different letters within a column indicate significant differences among the mean values (*p* < 0.05). PV: peak viscosity; BV: breakdown viscosity; FV: final viscosity; SV: setback viscosity; PT: pasting temperature. Data are the means of three replicates. N0, control; N150, nitrogen fertilizer; N0+B, biochar; N150+B, nitrogen fertilizer and biochar. ns, nonsignificant *p* < 0.05. ** *p* < 0.01.

**Table 7:** Thermal properties of starch. An analysis of variance (ANOVA) was utilized to assess the effects of treatment and variety interactions on the thermal properties of starch.

Varieties	Treatments	To (°C)	Tp (°C)	∆H (J/g)
SM1	N0	66.70 ± 0.36 bc	71.68 ± 0.09 c	15.94 ± 0.58 bc
	N150	68.26 ± 0.34 a	72.31 ± 0.22 b	18.26 ± 1.14 a
	N0+B	67.20 ± 0.27 b	72.99 ± 0.13 a	17.47 ± 0.46 ab
	N150+B	68.27 ± 0.55 a	72.07 ± 0.11 b	16.25 ± 0.95 bc
SM2	N0	65.75 ± 0.06 cd	69.82 ± 0.19 d	14.02 ± 0.98 d
	N150	66.31 ± 1.10b cd	69.74 ± 0.24 d	14.76 ± 0.47 cd
	N0+B	66.11 ± 0.38 cd	69.91 ± 0.15 d	15.04 ± 0.87 cd
	N150+B	65.42 ± 0.50 d	68.94 ± 0.07 e	14.25 ± 1.24 d
Analysis of variance	Varieties	**	**	**
	Treatments	*	**	**
	Varieties × Treatments	*	**	ns

Note: Different letters within a column indicate significant differences among the mean values (p < 0.05). Data are the means of three replicates. To, gelatinization onset; Tp, peak temperature; ∆H, gelatinization enthalpy. N0, control; N150, nitrogen fertilizer; N0+B, biochar; N150+B, nitrogen fertilizer and biochar. ns, nonsignificant. * *p* < 0.05. ** *p* < 0.01.

## Data Availability

The data used to support the findings of this study can be made available by the corresponding author upon request.
